# Laparoscopic feeding jejunostomy

**DOI:** 10.4103/0972-9941.62531

**Published:** 2010

**Authors:** Sourabh Aggarwal, Sahil Batra, Jesna S Mathew

**Affiliations:** Department of Surgery, University College of Medical Sciences, New Delhi, India

Sir,

We read the article ‘Novel cost-effective method of laparoscopic feeding-jejunostomy’[1] by Mistry *et al.* with interest. We appreciate the authors for coming out with an innovative simple cost-effective method of feeding jejunostomy by using regular laparoscopic instruments and an inexpensive readily available tube. It is indeed an achievement on their part.

We would like to add our views to the same. The indications for the surgery and morbidity status of the patients prior to the surgery are not mentioned which could have a great impact on the success of this innovative technique and on prognosis and complications in the patients. Despite only a single problem during the procedure, it has really shown impressive results. It is worth mentioning here that the results cannot be generalized at this stage. Full beneficence and side effect profile of such management can be realized only after long-term trials on a larger scale are undertaken. Nonetheless, the authors have done a commendable job and need to be applauded.
